# A Modified Electrochemical Sensor Based on N,S-Doped Carbon Dots/Carbon Nanotube-Poly(Amidoamine) Dendrimer Hybrids for Imatinib Mesylate Determination

**DOI:** 10.3390/bios13050547

**Published:** 2023-05-15

**Authors:** Maryam Saleh Mohammadnia, Hossein Roghani-Mamaqani, Masoumeh Ghalkhani, Salar Hemmati

**Affiliations:** 1 Faculty of Polymer Engineering, Sahand University of Technology, P.O. Box 51335-1996, Tabriz 533184-1133, Iran; 2 Institute of Polymeric Materials, Sahand University of Technology, P.O. Box 51335-1996, Tabriz 533184-1133, Iran; 3 Electrochemical Sensors Research Laboratory, Department of Chemistry, Faculty of Science, Shahid Rajaee Teacher Training University, Lavizan, P.O. Box 16785-163, Tehran 167881-5811, Iran; 4 Drug Applied Research Center, Tabriz University of Medical Sciences, Tabriz 516661-6471, Iran; 5 Biotechnology Research Center, Tabriz University of Medical Science, Tabriz 516661-6471, Iran

**Keywords:** imatinib mesylate, carbon nanotube, poly(amidoamine) dendrimer, N,S-doped carbon dots

## Abstract

Imatinib mesylate, an anticancer drug, is prescribed to treat gastrointestinal stromal tumors and chronic myelogenous leukemia. A hybrid nanocomposite of N,S-doped carbon dots/carbon nanotube-poly(amidoamine) dendrimer (N,S-CDs/CNTD) was successfully synthesized and used as a significant modifier to design a new and highly selective electrochemical sensor for the determination of imatinib mesylate. A rigorous study with electrochemical techniques, such as cyclic voltammetry and differential pulse voltammetry, was performed to elucidate the electrocatalytic properties of the as-prepared nanocomposite and the preparation procedure of the modified glassy carbon electrode (GCE). A higher oxidation peak current was generated for the imatinib mesylate on a N,S-CDs/CNTD/GCE surface compared to the GCE and CNTD/GCE. The N,S-CDs/CNTD/GCE showed a linear relationship between the concentration and oxidation peak current of the imatinib mesylate in 0.01–100 μM, with a detection limit of 3 nM. Finally, the imatinib mesylate’s quantification in blood-serum samples was successfully performed. The N,S-CDs/CNTD/GCE’s reproducibility and stability were indeed excellent.

## 1. Introduction

Cancer, in which cells in the body proliferate uncontrollably and then spread throughout different parts of the body, is a fatal disease [1]. One of the drugs confirmed by the Food and Drug Administration for cancer treatment is imatinib mesylate (IMAT). With molecular structure of 4-[(4-methylpiperazin-1-yl)methyl]a-N-[4-methyl-3-[(4-pyridin-3-ylpyrimidin-2-yl)amino]phenyl] benzamide, IMAT has been used to treat chronic melodic leukemia (CML) [2]. When this drug is administered, the activity of the tyrosine kinase enzyme, whose main function is to inhibit the Bcr-Abl fusion protein, is prevented, and the growth rate of cancer cells is reduced [3,4]. Specific dosages of IMAT lead to different concentrations in the bloodstreams of patients, which suggests a relationship between the concentration level of this drug and the clinical response [5]. Therefore, the progression of CML disease should be controlled by measuring the concentration of the IMAT drug at the plasma level. According to previous reports, monitoring IMB levels in real biological samples is of great environmental and clinical importance [6,7]. Various analyses, such as capillary electrophoresis, high-performance liquid chromatography, gas chromatography, and fluorescence spectroscopy, have been used to measure the concentration of IMAT [8,9,10,11,12,13]. Most of these methods, despite their high sensitivity and highly accurate measurements, are associated with limitations, such as high operating costs, long analysis responses, complex instrumentation, and the need for a large number of samples and skilled and capable operators. By contrast, electrochemical methods are cost-effective and sensitive, with short analytical responses and without the need for sophisticated instruments [14,15,16,17]. They involve the use of appropriate electrochemical sensors to perform the sensitive detection of electroactive substances [18,19,20]. Carbon electrodes have received more attention for use in electrochemical sensors than other electrodes due to their stability, non-reactivity with other materials, and vast electrochemical window [21,22,23,24]. Despite the noticeable features of carbon electrodes, they have a weak electrochemical signal, which is unsuitable for sensitive analyses. Therefore, the modification of carbon electrodes’ surfaces with potential materials will improve the electron transfer of the species in solution at the electrode surface, increase the electrocatalytic activity and, thus, improve the electrochemical responses of the electrode [25,26,27]. According to recent studies, the modification of the electrode surface with carbon nanomaterials, such as graphene, carbon nanotubes, graphene quantum dots, carbon quantum dots (CQDs), and carbon nanospheres, improves the electrochemical performance of the electrode [22,25,26,28,29,30].

Carbon dots (CDs), known as CQDs, are a new class of carbon-based nanomaterial with dimensions of less than 10 nm, which have been considered in many fields, such as electrochemical sensors, photocatalysis, and energy storage, due to their positive features, such as ease of synthesis, minor toxicity, high chemical stability, good dispersibility, biocompatibility, and outstanding optical properties [16,31,32,33,34]. Most of the early studies in this area were conducted on entirely carbon-containing CDs, and it was known that doping them with elements, such as nitrogen, sulfur, boron, and phosphorus, or their compounds, could be used to modulate their properties [35,36,37]. Nitrogen and sulfur atoms act as electron donors on the surfaces of CDs and, consequently, increase their ability to interact with various analyte species [38,39]. A small number of studies have been conducted on the use of CDs in electroanalysis. This intermittent use of CDs can be directly applied with the purpose of modifying electrodes alone or in combination with other compounds. Carbon dots are used in the electrochemical analysis of organic molecules more than other materials. In general, CDs may act as polyvalent redox species in these oxidative determinations, which can facilitate electron transfer and have catalytic benefits [40].

**Scheme 1:**
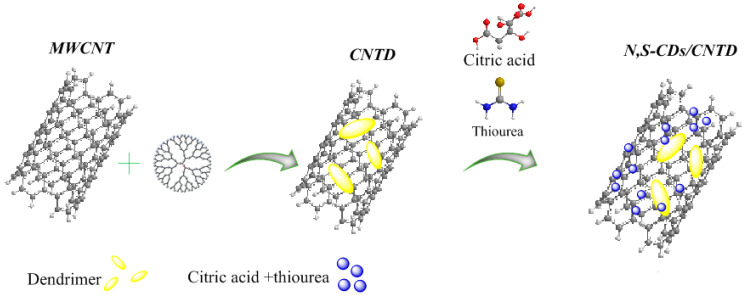
Schematic illustration of the synthesis of N,S-doped carbon dots/carbon nanotube-poly(amidoamine) dendrimer.

Chemical doping increases the density of charge carriers, resulting in increased electrical properties and catalytic activity. The combination of CDs with conductive nanomaterials, such as carbon nanotubes (CNTs) and graphene, is another solution to improve the dispersibility and electrocatalytic activity of these carbon nanomaterials [41,42,43,44]. Among carbon nanomaterials, CNTs have been considered in electrochemical sensors due to their outstanding optical, electrical, and mechanical properties, high charge-storage capacity, large surface area, excellent electrical conductivity, and chemical and mechanical stability [45,46]. One of the main issues with these nanomaterials is the ineffectiveness of their walls, which makes it difficult for other particles to deposit steadily and uniformly on their surface. The covalent or non-covalent functionalization of CNTs can improve the uniform distribution of nanoparticles on their surfaces, as well as improving their dispersibility in various solvents [47,48]. A suitable strategy for binding functional groups to CNT surfaces and enhancing their distribution and performance is the functionalization of CNT surfaces with nucleic acids, dendrimers, and polymers [16,49]. Multi-branched polymers or dendrimers with specific structures and architectures have been used to stabilize semiconductor nanoparticles [50]. In this study, a novel nanocomposite based on N,S-doped CDs/carbon nanotube-grafted poly(amidoamine) dendrimer (N,S-CDs/CNTD) was introduced as a potential modifier to construct an effective electrochemical sensor. Initially, carboxylic acid groups were created on the surfaces of the CNTs through interactions with nitric acid solution. Next, the CNT-NH_2_ structure was prepared through an amide reaction between the carboxylic acid and ethylene-diamine groups. Finally, the CNTD nanostructure was prepared through a two-step process, including (I) the preparation of the half-generation of dendrimer on a nanotube (CNTDG0.5) through the Michael-addition reaction between the amine groups on the CNT-NH_2_ and methyl acrylate, and (II) the synthesis of the first generation of dendrimer through the amidation between the CNTD0.5 and the ethylene diamine. Finally, as shown in Scheme 1, a one-pot synthesis using the hydrothermal method was used to prepare the CNTD decorated with N,S-CDs. In particular, thiourea, as a source of nitrogen and sulfur, can trap free citric acid on the surfaces and edges of CNTs.

When comparing different materials, the effect of the nitrogen-doped CDs was more noticeable than that of the sulfur-doped CDs, but the doping of the CNTD with both nitrogen and sulfur had better responses than that of the unmodified CNTD on the electrochemical sensors. On the other hand, in the case of the CNTD nanostructure, the spatial barrier created by the dendrimer separated the CNTs from each other and improved the electrode wettability in the solution. The CNTD acted as a conductive substrate for the CDs, facilitated electron transfer and prevented the accumulation of CDs, thereby providing more electroactive locations. The superb applicability of the sensor design was shown for the electrochemical measurement of the IMAT anticancer drug.

## 2. Experimental Methods

### 2.1. Materials

All chemicals, including multi-walled carbon nanotube (MWCNTs), methyl acrylate (MA), ethylenediamine (EDA), nitric acid (HNO_3_), tetrahydrofuran (THF), N,N′-dicyclohexylcarbodiimide (DCC), citric acid, and thiourea were obtained from Shenzhen Nanoport Co., China, Sigma-Aldrich, and Merck.

### 2.2. Synthesis of Modifiers

#### 2.2.1. Preparation of CNT-NH_2_

To create carboxylic acid groups on the surfaces of nanotubes, 100 mL of nitric acid (60%) was added to 2 g of MWCNTs and refluxed for 24 h. After the mixture was cooled to room temperature, it was filtered by a PTFE filter and rinsed several times with distilled water. Next, it was placed in an oven at 60 °C overnight. The CNT-NH_2_ was prepared via an amidation reaction between carboxyl groups of (CNT-COOH) and amino groups of ethylene diamine. First, 100 mL of THF was added to 4 g of nanotubes and stirred for 6 h, after which it was placed in an ultrasonic bath for 54 min. The mixture was added to a 250 round-bottomed flask, and after the addition of 0.5 g of DCC, 40 mL of ethylene diamine diluted with 40 mL of THF was added dropwise to the contents of the flask and stirred for 24 h. The reaction mixture was filtered through a PTFE-filter membrane and washed several times with THF. Finally, the black powder was dried at 60 °C for 24 h.

#### 2.2.2. Preparation of CNTD

This approach involved a two-step process, including (I) preparation of the half-generation of dendrimer on nanotube (CNTDG0.5) through the Michael-addition reaction between amine groups on CNTNH2 and methyl acrylate and (II) synthesis of the first generation of dendrimer through amidation between CNTD0.5 and ethylene diamine.

Step I: in total, 2 g of CNTNH_2_ was added to the 100 mL of THF and stirred for 10 h in a two-necked round-bottomed flask, followed by sonication for 45 min. Next, the mixture was purged with nitrogen gas and sealed. Subsequently, 6.4 mL of methyl acrylate in 20 mL of THF was added dropwise to the reaction at 0 °C in an ice bath and stirred for three days in a dark room. The final product was filtered through a 0.2-µm-filter membrane and washed with THF several times, and the black solid (CNTD-G0.5) was dried at 55 °C in oven for 24 h.

Step II-1: in total, 6 g of as-prepared CNTD-G0.5 black powder was stirred in a laboratory reactor containing 160 mL of THF for one day. After probe sonication for 45 min, the reactor was degassed with N_2_ and then sealed. Subsequently, the reactor was placed in the ice bath, 38 mL of EDA in 40 mL THF was added dropwise to the mixture, and stirring was continued for three days at room temperature. The mixture was filtered by a 0.22-μm filter membrane, and the black solid was washed several times with THF and dried in an oven at 55 °C to obtain the final product.

#### 2.2.3. Preparation of N,S-CDs/CNTD

The N,S-CDs/CNTD hybrid was prepared by the hydrothermal method. In brief, in a one-step synthesis, 0.03 g CNTD, 0.58 g citric acid, and 0.76 g thiourea were dispersed ultrasonically into 30.0 mL of double distilled water, successively. Next, the homogeneous mixture was transferred into a Teflon-lined hydrothermal autoclave (45 mL) and heated at 180 °C for 12 h. After this reaction, the autoclave was cooled to room temperature. The resultant sample was centrifuged at 100,000 rpm for 15 min and the sediment section of the sample containing N,S-CDs/CNTD was freeze-dried and stored at 4 °C.

### 2.3. Preparation of the Sensor

The glassy carbon electrode (GCE) was first polished using alumina powder. Next, the GCE surface was washed with water and ethanol, and after drying, it was activated by cyclic voltammetry technique in 0.1 M of sulfuric acid solution. Subsequently, 1 mg of the synthesized material was dispersed in 1 mL of DMF, and 2 μL of the material was placed on the electrode surface. After drying the electrode surface, IMAT measurement was performed.

### 2.4. Measurements and Analyses

All electrochemical analyses were performed using PSTrace software and a PalmSens EmStat3 + apparatus. The Fourier-transform infrared (FTIR) experiment was carried out in the spectral range of 400–4000 cm^−1^ using a PerkinElmer spectrometer. The X-ray diffraction (XRD) pattern was determined using a Siemens (Germany) X-ray diffractometer with Cu K_α_ radiation (λ = 0.1542 nm). The energy-dispersive X-ray spectroscopy (EDS) was carried out using a TESCAN MIRA3 SEM to confirm elemental composition of the samples. Elemental analysis (CHNS) was performed on a COSTEH elemental analyzer. The TEM images were obtained by a transmission-electron microscope (LEO 906 E, Germany) at an accelerator voltage of 100 kV. Photoluminescence (PL) spectra of CDs were measured with a JASCO FP-750 Spectrofluorometer.

## 3. Results

### 3.1. Characterization

**Figure 1:**
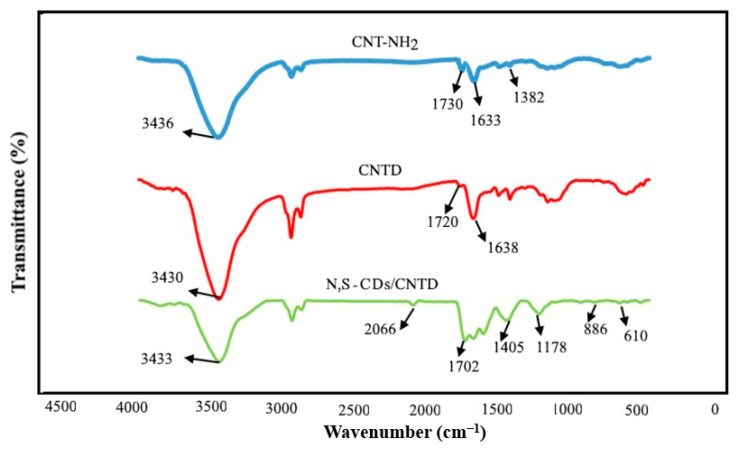
FTIR spectra of the CNT-NH_2_, CNTD, and N,S-CDs/CNTD.

The FTIR results of the CNT-NH_2_, CNTD, and N,S-CDs/CNTD are displayed in Figure 1. The CNTNH_2_ showed characteristic peaks at 1382 and 1633 cm^−1^, which were assigned to the stretching vibration of the C–N and the bending vibration of the N–H band, respectively. The stretching vibration of the C=O groups was at 1730 cm^−1^. Furthermore, the band observed at 3436 cm^−1^ was attributed to the stretching vibration of the N–H bands. In the case of the CNTD, the N–H bending vibration intensified at 1638 cm^−1^, and the stretching vibration of the carbonyl units of the methyl acrylate resulted in a peak at 1720 cm^−1^. The stretching vibration of the N–H units was also observed at 3430 cm^−1^. In the FTIR spectra of the N,S-CDs/CNTD, several characteristic peaks were identified. The sharp absorption peak at 3433 cm^−1^ corresponded to the stretching vibration of the N–H units. The stretching vibration of the C–H bond appeared at 2066 cm^−1^. The peak at 1702 cm^–1^ corresponds to the stretching vibration of the –C=O, and the peak at 1405 cm^–1^ was due to the C–N bending vibration. The C–OH stretching mode appeared at 1178 cm^–1^, and the peaks at 620 and 886 cm^–1^ corresponded to the C–S stretching vibration.

**Figure 2:**
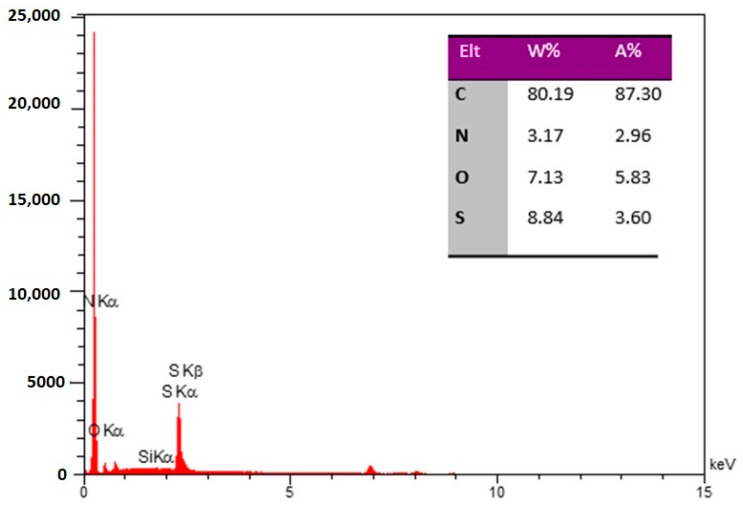
EDS spectrum of the N, S-CDs/CNTD sample.

The elemental analysis of the N,S-CDs/CNTD was also performed by EDS. The amount of elements detected by the EDS is shown in Figure 2. Carbon (80.19 wt%), nitrogen (3.17 wt%), oxygen (7.13 wt%), and sulfur (8.84 wt%) were identified on the surfaces of the hybrid nanoparticles. Therefore, according to the EDS data, the presence of nitrogen and sulfur in the N, S-CDs confirmed its successful formation. The results of the CHNS elemental analysis provide robust support for the structural formation of the prepared N,S-CDs. According to the CHNS results, the percentages of carbon, nitrogen, and sulfur in the N,S-CDs/CNTD were determined as 77.70, 3.32, and 5.40 wt%, respectively, which are close to the results of the EDS analysis.

**Figure 3:**
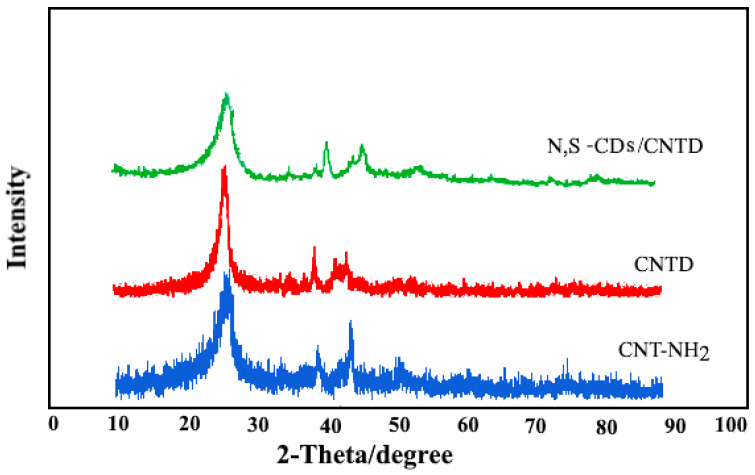
XRD patterns of CNT-NH_2_, CNTD, and N,S-CDs/CNTD.

Figure 3 shows the typical X-ray diffraction (XRD) spectra of the CNT-NH_2_, CNTD, and N, S-CDs/CNTD powders. In the CNT-NH_2_ pattern, a peak represented at the diffraction angle of 25° was related to the X-ray reflection of the plate (002). After the modification of the CNT-NH_2_, the diffraction angle of the prepared CNTD and N, S-CDs/CNTD decreased from 25.3 to 24.7° due to the increase in the distance between the layers of nanotubes during the functionalization. The other two peaks, at 2θ = 39.4 and 44.6°, were related to crystal plates (100) and (101), respectively.

**Figure 4:**
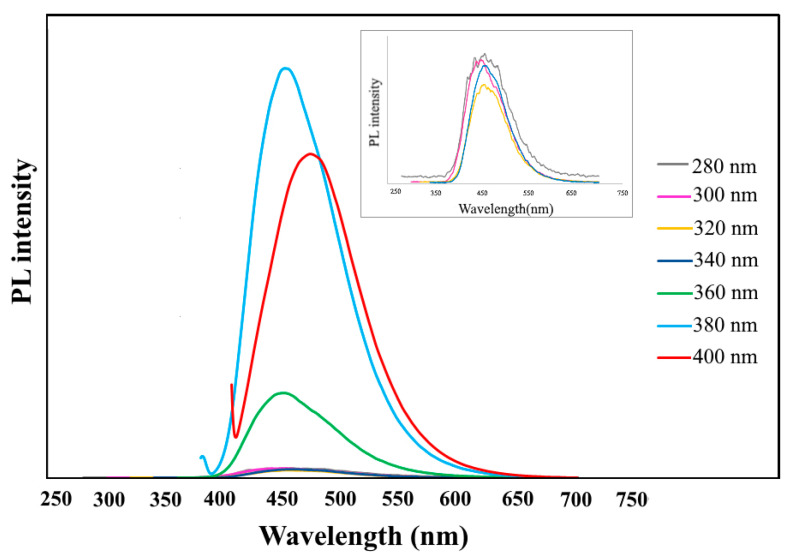
PL spectra of N,S-CDs under different excitation wavelengths.

For an accurate study of the N,S-CDs/CNTD, the PL measurements were performed using various excitation wavelengths, as shown in Figure 4. By changing the wavelength of excitation from 280 to 380 nm, the N,S-CDs/CNTD showed PL-excitation intensity at 450 nm, and the PL intensity was maximized at the excitation wavelength of 380 nm. In this case, the PL peaks at 450 nm were related to the n→π* transition of the carboxyl and carbonyl groups. Moreover, when the excitation wavelength increased from 380 to 400 nm, the location of the spectral emission peak of the N, S-CDs/CNTD red-shifted and its intensity decreased. It was found that the N, S-CDs/CNTD showed photoemission corresponding to the excitation wavelength due to changes in the distribution of the particle size. In addition, various functional groups containing nitrogen, oxygen, and sulfur atoms at the edge of the N, S-CDs might have generated a trap state between the levels of HOMO and LUMO.

**Figure 5:**
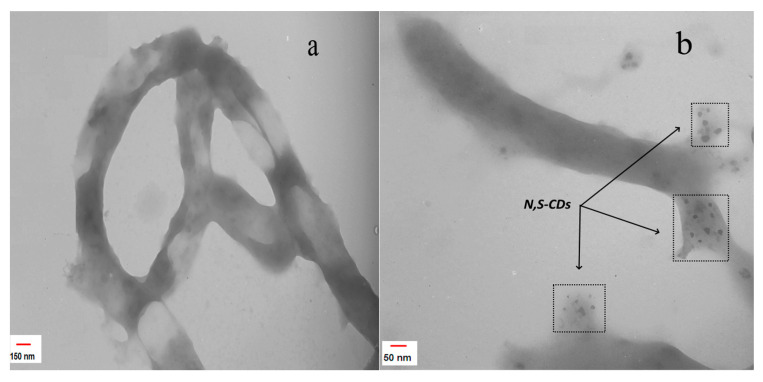
TEM images of the (**a**) CNTD and (**b**) N, S-CDs/CNTD hybrids.

The nitrogen and sulfur atoms doped on the surfaces of the CDs acted as electron donors and increased their sensitivity to interactions with various analytical species [51]. To investigate the origin of these properties, the TEM analysis was performed to determine the size, morphology, and structure of the synthesized nanoparticles. Figure 5a,b show the representative TEM images of the CNTD and N, S-CDs/CNTD hybrid nanocomposites, respectively. Figure 5a shows carbon-nanotube surfaces grafted with layers of PAMAM dendrimers, which indicated the formation of the CNTD nanohybrids. Figure 5b demonstrates the formation of small-sized monodispersed N,S-CDs on dendrimer-modified carbon nanotubes.

### 3.2. Electrochemical Analyses

**Figure 6:**
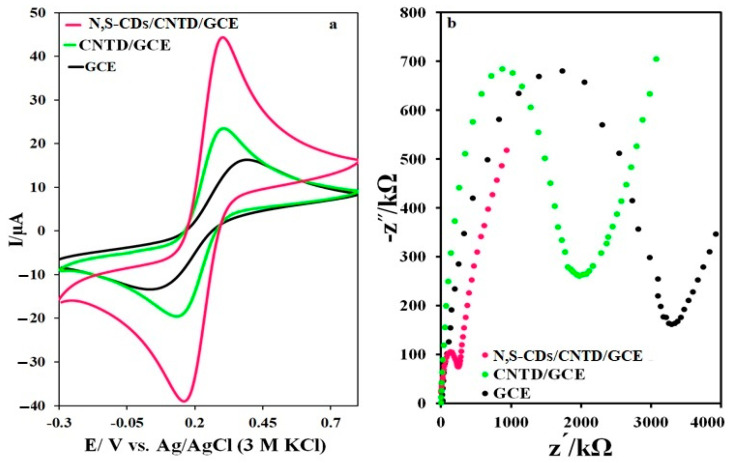
(**a**) CVs and (**b**) Nyquist plot of the GCE, CNTD/GCE, and N,S-CDs/CNTD/GCE in a 1-mM electrochemical probe solution.

The performance of the prepared electrodes in a probe solution containing 1 mM K_4_[Fe(CN)_6_]/K_3_[Fe(CN)_6_] and 0.1 M KCl was investigated by the cyclic voltammetry method, and the results are shown in Figure 6a. The differences between the oxidation and reduction peaks’ potential at GCE, CNTD/GCE, and N,S-CDs/CNTD/GCE were 0.35, 0.17, and 0.13 V, respectively. The oxidation current of the electrochemical probe at the surface of the N,S-CDs/CNTD/GCE was greater than that of the GCE and CNTD/GCE, which indicates that the N,S-CDs/CNTD/GCE had better electrocatalytic properties. The electrochemical impedance spectroscopy results confirmed the better electrochemical performance of the N,S-CDs/CNTD/GCE. Figure 6b shows the Nyquist diagram of the GCE, CNTD/GCE, and N,S-CDs/CNTD/GCE. The charge-transfer-resistance values of the GCE, CNTD/GCE, and N,S-CDs/CNTD/GCE were 4.43, 1.25, and 0.43 Ω, respectively. The lower resistance on the surface of the N,S-CDs/CNTD/GCE indicates its better electrochemical behavior [52]. The surface area of the GCE, CNTD/GCE, and N,S-CDs/CNTD/GCE was calculated using the Randles–Sevcik equation. The area obtained from the electrochemical response of the N,S-CDs/CNTD/GCE was greater than that obtained with the other electrodes, indicating its good capability as an electrochemical sensor.

**Figure 7:**
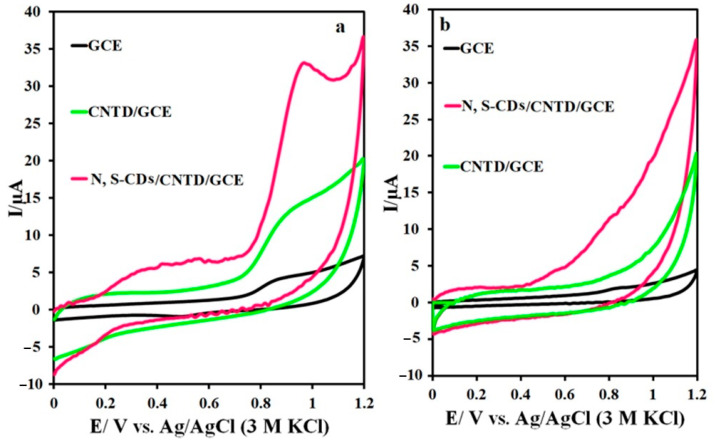
(**a**) CVs of the GCE, CNTD/GCE, and N,S-CDs/CNTD/GCE in the (**a**) presence and (**b**) absence of IMAT.

The electrochemical behavior of the IMAT on the surfaces of the GCE, CNTD/GCE, and N,S-CDs/CNTD/GCE was also investigated (Figure 7a). The oxidation potential of the IMAT on the GCE was 0.97 V without any reduction peaks, indicating an irreversible reaction. The oxidative peak current of the IMAT was equal to 2 μA on the GCE, which was about six and sixteen times less than the CNTD/GCE and N,S-CDs/CNTD/GCE, respectively. The oxidation-peak potential of the IMAT on the CNTD/GCE and N,S-CDs/CNTD/GCE was 0.93 V. Notably, the presence of dendrimer-containing CNTs improved the electrodes’ conductivity and extended their effective surface area. The synergistic effect of the N,S-CDs and CNTD facilitated the electron-transfer rate of the IMAT oxidation process on the N,S-CDs/CNTD/GCE and provided perfect capability for the IMAT analysis. No peaks were observed in the absence of the drug on the GCE, CNTD/GCE, or N,S-CDs/CNTD/GCE (Figure 7b).

**Figure 8:**
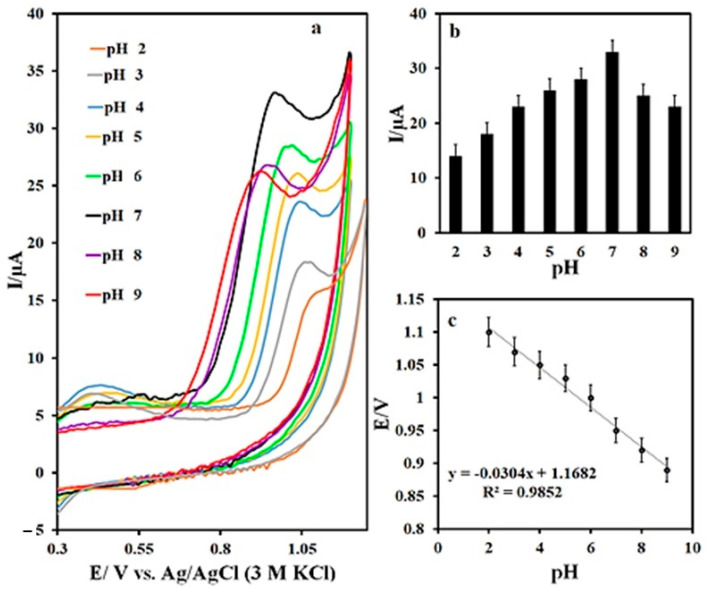
(**a**) CVs of IMAT at different pH, (**b**) plot of *I*_pa_ against pH, and (**c**) plot of *E*_pa_ against pH.

An investigation of the effect of the pH of the buffer solution on the voltammetric response of the IMAT revealed effective pH behavior. Figure 8a shows the IMAT cyclic voltammograms at N,S-CDs/CNTD/GCE. Accordingly, the oxidation-peak potential of the IMAT shifted to negative values with increasing pH. The maximum peak current was related to pH 7, so this pH value was used as the optimum condition for further evaluations (Figure 8b). In addition, the peak-potential curve versus the pH for the IMAT oxidation process showed a slope value of −0.03 V/pH, which pointed to the double contribution of the electrons and protons in the process of IMAT oxidation (Figure 8c).

**Figure 9:**
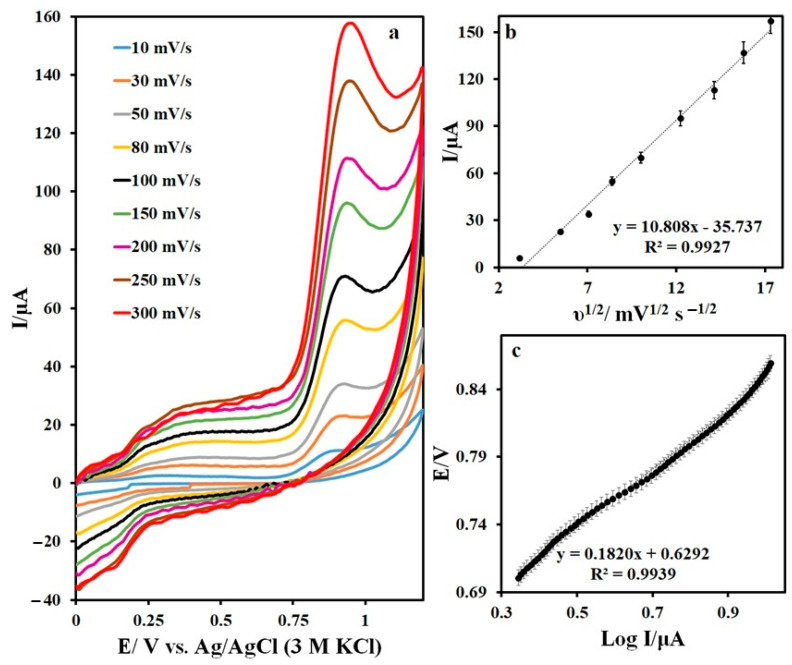
(**a**) CVs of IMAT at different scan rates, (**b**) plot of *I*_pa_ against square of scan rate, and (**c**) plot of *E*_pa_ against Log *I*_pa_.

The scanning rate of the IMAT oxidation is shown in Figure 9a. The evaluation of the peak currents of the IMAT cyclic voltammograms recorded on the N,S-CDs/CNTD/GCE in a buffered solution (pH 7) revealed a linear relationship between the peak current and the square root of the potential scan rate, indicating a diffusion-controlled reaction for the IMAT oxidation (Figure 9b). The electron-transfer coefficient (α) was obtained by plotting the potential curve against the logarithm of the peak current at a scan speed of 10 mV/s (Figure 9c). The α = 0.63 was calculated by considering the slope value (0.1820) of the plotted curve, which was equal to 2.303 RT/(1-α)nF.

**Figure 10:**
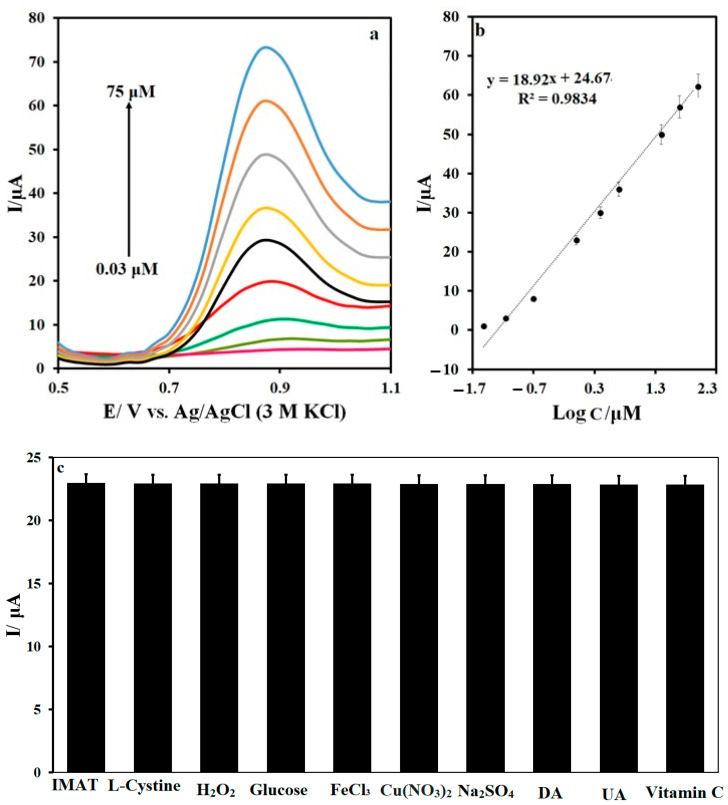
(**a**) DPVs of different concentrations of IMAT, (**b**) the calibration curve (**c**), and the peak current of 5-μM IMAT recorded in the presence of 100 μM of some potential interferences.

Different concentrations were measured for the N,S-CDs/CNTD/GCE at pH 7.0 by using the differential pulse voltammetry technique, as shown in Figure 10a. The linear relationship of the peak current with the IMAT concentration was obtained in the concentration range of 0.03–75 Μm (Figure 10b). The detection limit of the IMAT by the N,S-CDs/CNTD/GCE was calculated as LOD = 3 s/m equal to 0.01 μM. A comparison of the IMAT-measurement performance in previously reported methods with that of the N,S-CDs/CNTD/GCE sensor is given in Table 1. The results show that the N,S-CDs/CNTD/GCE produced a comparable LOD for IMAT analysis to the other methods and a more expansive concentration range. Therefore, the N,S-CDs/CNTD/GCE has outstanding capability in IMAT analysis.

The reproducibility of the N,S-CDs/CNTD/GCE was evaluated by five consecutive measurements of the IMAT concentration. The results showed that the relative standard deviation obtained from the IMAT oxidation peak currents after five repetitions was equal to 3.45, which indicates the appropriate reproducibility of the N,S-CDs/CNTD/GCE. The stability test of the N,S-CDs/CNTD/GCE for 30 days showed that the IMAT’s peak current changed by about 2.4% by the thirtieth day compared to its value of the first day of the experiments. This indicates that the modified electrode had very good stability. The electrochemical measurements of the IMAT were investigated in the presence of various compounds using N,S-CDs/CNTD/GCE, as shown in Figure 10c. The results showed a slight change in the IMAT’s oxidation peak current in the presence of the evaluated compounds. Considering that the observed changes were less than 5%, it can be concluded that the N,S-CDs/CNTD/GCE has an appropriate selectivity for IMAT measurements. The IMAT measurement on the N,S-CDs/CNTD/GCE in the human-plasma samples was performed by using the standard addition method. The results are given in Table 2. After the standard addition of IMAT to the blood sample at 1, 3, and 10 μM, recovery percentages of about 109–92% were obtained when performing an analysis of the N,S-CDs/CNTD/GCE. These results confirmed the good ability of the N,S-CDs/CNTD/GCE to measure IMAT in a real sample.

## 4. Conclusions

An IMAT measurement was performed on the surface of N,S-CDs/CNTD/GCE. The N, S-CDs modifier composited with CNTD expanded the effective surface area of the sensor, and the conductivity and wettability of the electrode surface were boosted by the CNTD. On the other hand, the presence of CDS containing nitrogen and sulfur, which improved the performance of the carbon dot, showed very good results for IMAT analysis. The low detection limit, high selectivity, and sensitivity were among the most important features of this electrochemical sensor for measuring IMAT. In addition, the high interference tolerance of N,S-CDs/CNTD/GCE when measuring IMAT in real examples is an appreciable feature.

## Figures and Tables

**Table 1:** Comparison of the performances of different analytical methods for IMAT measurement.

Modifier	Method	Liner Range (μM)	LOD (μM)	Ref.
C18	LC-UV	1–40	0.11	[53]
-	HPTLC	0.2–20	0.02	[54]
-	GC-MS	0.02–0.2	0.002	[55]
C18	UHPLC	0.1–72	0.02	[56]
MWCNT/SPCE	SWV	50–912	16	[57]
N,S-CDs/CNTD/GCE	DPV	0.03–75	0.01	Our work

**Table 2:** The measurement of IMAT in human-blood serum.

Add (μM)	Obtained (μM)	Recovery (%)	RSD (%)
1	0.92	92%	4.2%
5	4.85	97%	4.1%
10	9.78	97%	4.1%

## Data Availability

Not applicable.
